# The World Psychiatry Exchange Program: insights from Tunisians in India

**DOI:** 10.1192/bji.2024.21

**Published:** 2024-11

**Authors:** Mona Daoud, Wafa Abdelghaffar, Philip Sharad, Ramdas Ransing, Mariana Pinto da Costa

**Affiliations:** 1 Assistant Professor of Child and Adolescent Psychiatry, Mongi Slim Hospital, La Marsa, Tunisia. Email: mona.daoud@fmt.utm.tn; 2 Assistant Professor of Psychiatry, Mongi Slim Hospital, La Marsa, Tunisia; 3 Assistant Professor, Department of Psychiatry, Clinical Neurosciences and Addiction Medicine, All India Institute of Medical Sciences (AIIMS), Guwahati, Assam, India; 4 Associate Professor, Department of Psychiatry, Clinical Neurosciences and Addiction Medicine, All India Institute of Medical Sciences (AIIMS), Guwahati, Assam, India; 5 Consultant Psychiatrist, Institute of Psychiatry, Psychology & Neuroscience, King's College London, London, UK

**Keywords:** World Psychiatry Exchange Program, early career psychiatrists (ECPs), child psychiatry, Tunisia, India

## Abstract

The World Psychiatry Exchange Program offers opportunities overseas for early career psychiatrists (ECPs), fostering immersion in clinical and cultural contexts. In this article, we present the experiences of two Tunisian ECPs in India. Activities included observing interviews, and attending courses and webinars. Challenges and opportunities in perinatal psychiatry and in child psychiatry were observed, emphasising cross-cultural nuances. Language barriers were overcome through translation. Notably, collaboration and proximity between departments countered mental illness stigma among medical professionals. This exchange underscores the importance of cultural awareness, collaboration and contextual adaptation in psychiatry. Lessons from this cross-cultural experience offer insights for enhanced care and research in diverse settings. This exchange also allowed for a rich scientific and cultural experience and brought to light many commonalities between India and Tunisia.

Every year the Early Career Psychiatrists (ECPs) Section of the World Psychiatric Association offers ECPs an opportunity to participate in short exchanges overseas, as part of the World Psychiatry Exchange Program.^1^ It allows participants an immersive experience in both the clinical practice and the culture of the host country. One of the strong points of this programme is that the choice of the focus of the exchange is entirely up to the two parties involved. Two ECPs from Tunisia (M.D. and W.A.) took part in the exchange programme to obtain insights into psychiatric practice for adults and children in a context different from their own, with a specific emphasis on women's health and perinatal psychiatry. This article presents their experiences in the psychiatry department at the All India Institute of Medical Sciences (AIIMS) in Guwahati, Assam, India.

## Programme activities

The AIIMS Guwahati, Assam is the most recent facility of the AIIMS organisation, which is of national importance in India. It serves a dual purpose, functioning as a medical school with student accommodation and as a multidisciplinary teaching hospital with in-patient and out-patient units. This arrangement facilitated participation of the two ECPs in both clinical and academic activities.

Although the exchange was supervised by faculty members from the psychiatry department, there was a remarkable level of interdisciplinary collaboration. The organisational framework and policies enacted at AIIMS Guwahati facilitate such collaboration. For example, all medical doctors are invited to attend daily interdisciplinary critical paper readings. The proximity of various departments within the same building facilitates communication between different specialists. Notably, the psychiatry department is located only a few metres away from the paediatrics and obstetrics and gynaecology (OB-GYN) department, which makes referring patients and sharing crucial information among colleagues easier. Communication is facilitated thanks to a common internet Wi-Fi connection within the facility. Overall, the attitudes of different doctors towards mental health and psychiatry were remarkably positive. Unfortunately, literature has shown that stigmatising attitudes towards psychiatry and mental illness remain pervasive among doctors in India, Tunisia and around the world.^2–4^

In this exchange, scientific and clinical activities were organised according to a daily schedule. These activities encompassed observing psychiatric interviews with both adult and paediatric patients with faculty members of the psychiatry department and general paediatrics department, which were translated into English. The two ECPs were also able to directly interview some English-speaking patients. They attended a psychiatry lecture for first-year students delivered by an assistant professor of psychiatry (P.S.). Two webinars were organised in collaboration with colleagues from India, Tunisia, Thailand and Indonesia.^5^ Meetings with faculty from community and family medicine and forensic medicine took place to discuss topics such as mental health, child sexual abuse and domestic violence, with plans for future research projects. One of the most enriching experiences of the exchange was a community visit to a rural health sub-centre, including a home visit to a post-partum woman and her 6-month old baby. This visit was conducted by an assistant professor of community and family medicine, and medical students and offered a unique first-hand experience of perinatal care in an authentic local setting. A visit to the OB-GYN department took place, during which faculty discussed perinatal mental health, followed by a visit to the hospital's HIV screening unit for pregnant women.

## Child psychiatry in India and Tunisia

Regarding child psychiatry training, there are several differences in the training programmes between the two countries. In Tunisia, child and adolescent psychiatry is a separate medical specialty with a 5-year training programme including 1 year of adult psychiatry training. In India, child and adolescent psychiatry is a specialty of psychiatry that requires an additional 3 years of training after completing general psychiatry training, giving 6 years of training in total. The 3-year-long general psychiatry training includes rotations in different specialties, such as child and adolescent psychiatry and addiction medicine. In addition, some academic institutes (universities, professional associations) in India have started 1-year fellowship programmes to address the existing shortage of child and adolescent psychiatrists and perinatal psychiatrists.^6^

The role of child psychiatrists in perinatal care is loosely defined within biopsychosocial models of perinatal care.^7^ Conceptually, perinatal psychiatry adopts a dual approach: a medical perspective centred on mental disorders affecting the mother during the perinatal period and the potential risk of transmission to the child; and a developmental psychodynamic approach focused on the impact of dysfunction in the mother–child dyad – whether triggered by mental illness or not – on the newborn's development and transgenerational risk. Although care provided by psychiatrists would have a curative aim, child psychiatrists would be more focused on prevention. This dual approach is delineated by differences in perinatal care models between the UK and France. The British approach focuses on psychiatric disorders in the parent and the impact of the disorder on the child and the parent–child relationship. Conversely, the French approach scrutinises parent–child interaction from a psychoanalytic and developmental perspective in terms of risks for the child's development.^7^ Considering the historical and cultural influence exerted by these two countries on India and Tunisia respectively, during the colonial period and beyond, it is important to take these different approaches into account when conceptualising perinatal mental healthcare in the Indian and Tunisian contexts. It is the authors’ impression that, in both countries, psychiatrists and child psychiatrists are building a rather eclectic approach, that tries, primarily, to respond to the needs of local populations.

## Opportunities and challenges

Given the importance of verbal communication in psychiatry, the language barrier could have been a serious impediment to benefitting from this exchange. However, the interviews were not only translated into English for the ECPs, but also into Hindi for the Indian hosts (P.S. and R.R.), from the local Assamese language by a nurse. Coming from a fairly homogeneous Arabic-speaking linguistic landscape, the adaptability of both healthcare workers and patients in India was truly remarkable.

In spite of considerable cultural, linguistic and religious diversity in the Indian context, it was the unifying points that had most impact on everyday interactions in the hospital. Several commonalities were identified, akin to those observed in Tunisia, such as attitudes towards mental illness and stigma, as well as the prevalence of the traditional family structure in society.

Regarding women's mental health, interactions with faculty, nursing staff and patients highlighted some challenges faced by Indian women, such as dowry customs, social imperatives to marry from one's cast and religion, stigma towards divorced women, domestic violence and misogyny. These cultural facets might play a role in amplifying the mental illness burden among women.^8^

Concerning perinatal care, shared elements were the strong perinatal health programmes focused on community medicine and first-line healthcare started in the 1990s, with an increasing emphasis on mental health.^5^

## Conclusions

Even though drawing comparisons between India and Tunisia in various aspects might appear challenging, especially considering factors such as scale (population, land mass, resources), our experience in mental health services has identified numerous similarities. The presence of common cultural and historical elements between Tunisia and India establishes a good basis for future collaboration in the domain of mental health.

## Data Availability

Data availability is not applicable to this article as no new data were created or analysed in this study.
